# CSF extracellular vesicle proteomics demonstrates altered protein homeostasis in amyotrophic lateral sclerosis

**DOI:** 10.1186/s12014-020-09294-7

**Published:** 2020-08-17

**Authors:** Alexander G. Thompson, Elizabeth Gray, Imre Mäger, Marie-Laëtitia Thézénas, Philip D. Charles, Kevin Talbot, Roman Fischer, Benedikt M. Kessler, Mathew Wood, Martin R. Turner

**Affiliations:** 1 Nuffield Department of Clinical Neurosciences, University of Oxford, Level 6, West Wing, John Radcliffe Hospital, Oxford, OX3 9DU UK; 2 Department of Paediatrics, University of Oxford, Le Gros Clark Building, South Parks Road, Oxford, OX1 3QX UK; 3 Target Discovery Institute, Nuffield Department of Medicine, University of Oxford, Oxford, OX3 7FZ UK

**Keywords:** Amyotrophic lateral sclerosis, Exosome, Extracellular vesicle, CSF, Biomarker

## Abstract

**Background:**

Extracellular vesicles (EVs) released by neurons and glia reach the cerebrospinal fluid (CSF). Studying the proteome of CSF-derived EVs offers a novel perspective on the key intracellular processes associated with the pathogenesis of the neurodegenerative disease amyotrophic lateral sclerosis (ALS) and a potential source from which to develop biomarkers.

**Methods:**

CSF EVs were extracted using ultrafiltration liquid chromatography from ALS patients and controls. EV size distribution and concentration was measured using nanoparticle tracking analysis and liquid chromatography-tandem mass spectrometry proteomic analysis performed.

**Results:**

CSF EV concentration and size distribution did not differ between ALS and control groups, nor between a sub-group of ALS patients with or without an associated hexanucleotide repeat expansion (HRE) in *C9orf72*. Univariate proteomic analysis identified downregulation of the pentameric proteasome-like protein Bleomycin hydrolase in ALS patients, whilst Gene Ontology enrichment analysis demonstrated downregulation of proteasome core complex proteins (8/8 proteins, normalized enrichment ratio -1.77, FDR-adjusted *p* = 0.057) in the ALS group. The sub-group of ALS patients associated with the *C9orf72* HRE showed upregulation in Ubiquitin-like modifying-activating protein 1 (UBA1) compared to non-*C9orf72* cases.

**Conclusions:**

Proteomic analysis of CSF EVs in ALS detects intracellular alterations in protein homeostatic mechanisms, previously only identified in pathological tissues. This supports the wider use of CSF EVs as a source of novel biomarkers reflecting key and potentially druggable pathological intracellular pathway alterations in ALS.

## Introduction

The pathogenesis of the neurodegenerative disease amyotrophic lateral sclerosis (ALS) implicates an expanding range of cellular pathways [1], for which biomarkers are a pressing need [2]. Most cases of ALS are sporadic, though around 10% are attributable to mutation in one of a handful of genes, the most common of which is a hexanucleotide repeat expansion in an intronic region of the *C9orf72* gene [3]. Extracellular vesicles (EVs), including microvesicles and exosomes, are a heterogeneous population of membrane-bound structures ranging from 50 to 1000 nm in diameter released from cells into the extracellular milieu by a diversity of cell types [4]. A proportion of those released from central nervous system cells ultimately appear in the cerebrospinal fluid (CSF) [5, 6].

CSF EVs have unique potential to provide insight into intracellular processes beyond that of studying the whole CSF proteome, which contains largely secreted proteins [5]. This offers the prospect of revealing the key cellular perturbations underlying ALS, making CSF EVs an attractive potential source of biomarkers. EVs have been implicated in the pathogenesis ALS, predominantly as a potential mediator of intercellular spread of misfolded TDP-43 [7], inclusions of which are found in over 95% of ALS cases, including those with a *C9orf72* hexanucleotide repeat expansion [8]. Beyond putative spreading of pathology in prion-like propagation models of ALS, alterations in EV secretion may have a core role in ALS pathogenesis through defects in their role in the disposal of unwanted proteins and intercellular transport [9]. Inhibition of exosome biogenesis leads to increases in insoluble TDP-43 in a TDP-43-mutant mouse model, and reduced EV secretion is observed in fibroblasts and iPSC-derived motor neurons from patients carrying the *C9orf72* hexanucleotide repeat expansion [10, 11]. Although TDP-43 was also found at elevated levels within EV extracted from brain of patients with ALS in one report [10], studies examining the quantities of TDP-43 in CSF EVs from ALS and FTD patients have given conflicting results, with the largest study suggesting no difference compared with controls, potentially due to the diverse origin of CSF EV and the ubiquity of TDP-43 expression [12, 13].

The paucity of EVs within CSF, however, makes biomarker discovery experiments using agnostic methods such as proteomics challenging. In consequence, few studies have yet taken this approach [14]. This study used an optimised high-yield, high-purity CSF EV extraction method combined with quantification and subsequent proteomic analysis of EVs extracted from CSF of patients with ALS and controls to identify novel candidate biomarkers of ALS.

## Materials and methods

**Fig. 1:**
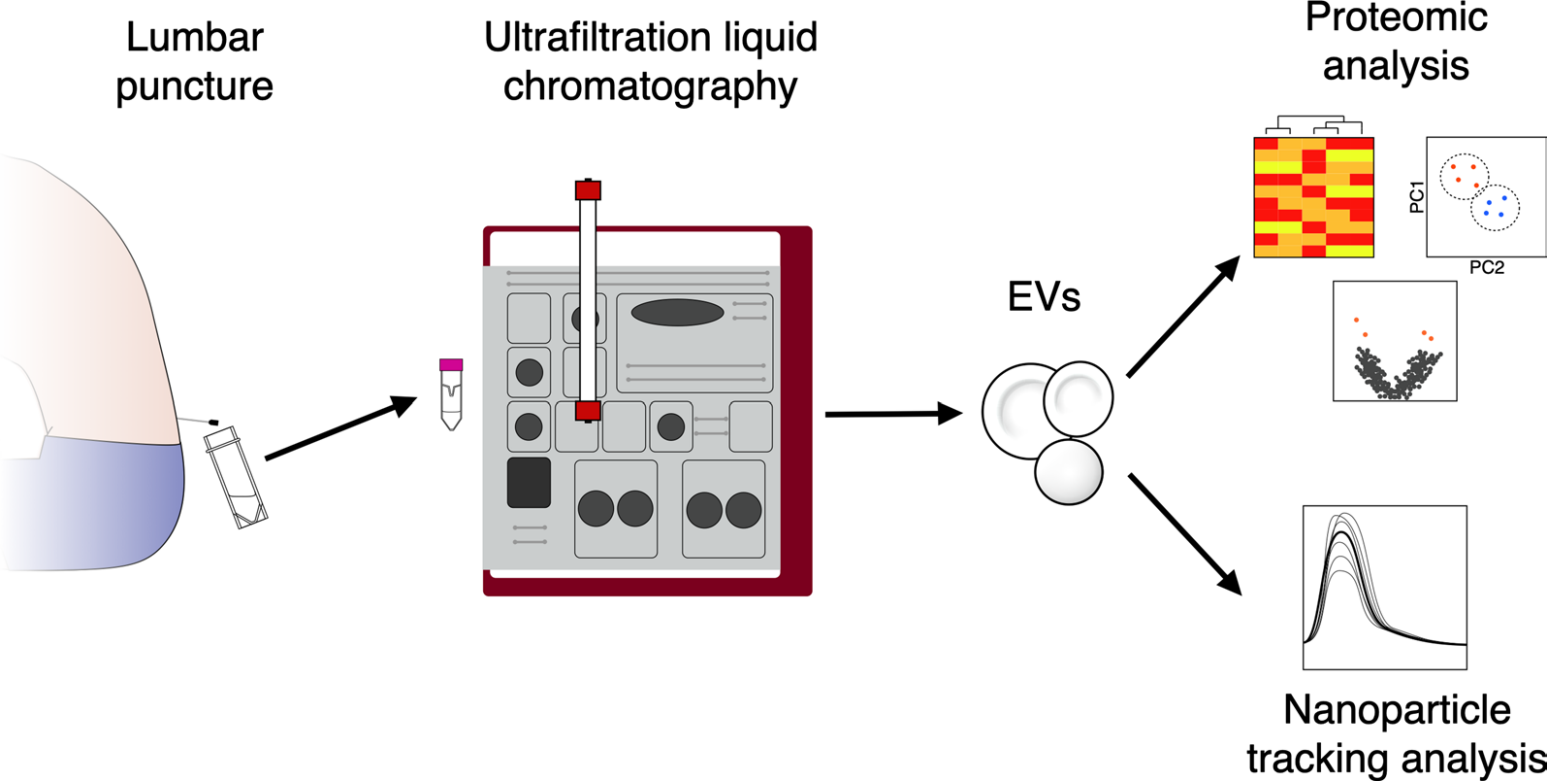
Experimental workflow. CSF samples obtained by lumbar puncture from patients with ALS and healthy controls were stored at – 80 °C until use. EVs were extracted from patient CSF samples using ultrafiltration liquid chromatography, with resulting EV samples subjected to liquid chromatography-tandem mass spectrometry proteomic analysis with label-free quantification, with subsequent multivariate, univariate and pathway analysis. ALS: amyotrophic lateral sclerosis; CSF: cerebrospinal fluid; EV: extracellular vesicle

The overall experimental workflow is outlined in Fig. 1.

### Participants and sampling

Consecutive individuals attending a tertiary referral clinic run by two experienced neurologists (KT, MRT), were offered participation after being diagnosed with ALS (n = 20) or as accompanying non-related healthy controls (n = 9). Four ALS patients were carrying a pathological hexanucleotide repeat expansion in *C9orf72* (Table 1). Ethical approval for the study was obtained from NRES Central Committee South Central—Berkshire (14/SC/0083). All participants provided written consent (or gave permission for a carer to sign on their behalf). Date of reported first symptom was taken as onset date and the rate of disease progression calculated using the revised ALS functional rating scale (ALSFRSR) as (48-ALSFRSR)/[time from symptom onset (months)].

**Table 1:** Demographic data for the CSF EV cohort

	ALS			Healthy control	*p*	
	All	*C9orf72*+	*C9orf72*−		HC vs ALS	*C9orf72 *+ *vs* non-*C9orf72*-
N	20	4	16	9	–	–
%male	75	50	81	55.6	0.396^a^	0.248^a^
Age at sampling, mean ± SD (years)	60.0 ± 10.9	53.0 ± 12.9	61.8 ± 10.1	54.5 ± 12.6	0.258^b^	0.186^b^
Age at onset, mean ± SD (years)	57.8 ± 10.7	52.1 ± 12.9	59.2 ± 10.2	–	–	0.014^b^
% bulbar onset	25.0	50.0	12.5	–	–	0.136^a^
Disease progression rate, median [IQR]	0.36 [0.18–0.58]	0.47 [0.26–0.73]	0.33 [0.18–0.55]	–	–	0.57^b^

*ALS* amyotrophic lateral sclerosis, *HC* healthy control, *SD* standard deviation, *IQR* interquartile range

^a^Fisher exact test, ^b^Kruskal–Wallis *H* test

CSF samples were obtained by lumbar puncture directly into polypropylene collection tubes. Samples were centrifuged at 3000 rpm for 10 min at 4 °C within 1 h of sampling and stored at − 80 °C until EV extraction.

### EV extraction

CSF samples were excluded if they had a red blood cell count > 200/mm^3^ or visible blood staining. Sample order was randomised prior to EV extraction. EVs were isolated from 7.2 mL CSF. CSF EV extraction and EV characterisation was performed as previously described [5]. CSF underwent centrifugation at 1200*g* for 10 min and was then filtered through a 0.22 μm Millex 33 mm polyetherosulfone syringe‐driven filter (Merck Millipore). Samples were filtered using Amicon Ultra‐15 100 kDa molecular weight cut-off (MWCO) centrifugal filters (Merck Millipore) at 3500*g* for 8 min, washed with 4 mL PBS and centrifuged at 3500*g* for 4 min. Retentate volume was adjusted to 800 μL with PBS, injected into a 24 mL size exclusion column packed with Sepharose 4 fast flow (mean particle size 90 μm, exclusion limit M_r_ 3 × 10^7^) and eluted with 40 mL PBS at 0.5 mL/min using an ÄKTA pure chromatography system (GE Life Sciences). Two mL fractions were collected from 6 to 40 mL elution volume. EV‐containing fractions [2, 3] were concentrated for further analysis using Amicon 10 kDa MWCO 4 mL centrifugal filters at 3500*g* to a retentate volume of < 400 μL.

### EV characterisation

EV size distribution and concentration was ascertained in fractions 2 and 3 by Nanoparticle Tracking Analysis (NTA) using a NanoSight NS500 (Malvern Panalytical, UK) and NTA 2.3 software. Where necessary, samples were diluted in PBS to achieve a concentration of 2 × 10^8^–2 × 10^9^ particles per mL. The camera level was set to 14 and detection threshold 5. Three recordings of 30–60 s were obtained for each sample and estimations of size distribution were averaged across recordings. EV marker and contaminating proteins were selected according to the 2018 International Society for Extracellular Vesicles position paper [15]. Transmission electron microscopy of a pooled sample of extracted control CSF EVs. 10 µL of extracted CSF EVs was applied to freshly glow‐discharged carbon‐coated 200 mesh copper grids for 2 min, blotted with filter paper, and stained with 2% uranyl acetate for 10 s, blotted and air dried. Grids were imaged in a FEI Tecnai 12 transmission electron microscope at 120 kV using a Gatan OneView CMOS camera.

### Sample preparation for proteomic analysis

Samples were reduced in 5 mM DTT for 30 min at room temperature followed by alkylation with 20 mM IAA for 30 min at room temperature. Samples were subsequently precipitated using chloroform–methanol precipitation. Precipitated protein was resuspended in 50 mM triethyl ammonium bicarbonate (TEAB) with vortexing and sonication for 2 min. Samples were digested overnight at 37 °C at 300 rpm using 400 ng of trypsin. Peptide digests were acidified with 1% formic acid and desalted using SOLA SPE cartridges (Thermofisher Scientific, UK) and dried by vacuum centrifugation. Peptide samples were resuspended in 10 μL of buffer A (2% acetonitrile, 0.1% formic acid in water). The entire sample of extracted EV peptide was injected for analysis by LC–MS/MS. Sample order was randomised prior to MS analysis and a sample produced by pooling an equal volume of peptide from each sample was run after every 10 samples.

### Liquid chromatography-tandem mass spectrometry

Peptides were analysed by nUHPLC LC–MS/MS using a Dionex Ultimate 3000 nanoUPLC, (Thermofisher Scientific, Germany) coupled to an Orbitrap Fusion Lumos Tribrid mass spectrometer (Thermo Scientific, Germany). Samples of peptide digest were loaded onto an EASY-Spray column (75 mÅ–500 mm, 2 μm particle size, ThermoFisher Scientific, Germany) and eluted using a 60-min gradient starting with 2% acetonitrile with 0.1% formic acid and 5% DMSO and increasing to 35% acetonitrile with 0.1% formic acid and 5% DMSO at a flow rate of 250 nL/min. The data were acquired in data dependent mode with a resolution of 120,000 full-width half maximum at m/z 200 in the survey scan (375–1500 m/z) and with EASY-IC using the reagent ion source (202 m/z) for internal calibration. MS/MS spectra were acquired after precursor isolation in the quadrupole with an isolation window of 1.2 Th, dynamic precursor selection (top speed mode) with a fixed duty cycle time of 3 s and dynamic precursor exclusion of 60 s. Isolated precursor ions were fragmented by CID with a normalised Collision Energy of 35%. Parallelization was enabled and MS/MS spectra were acquired in the linear ion trap for up to 250 ms with an ion target of 4000 in rapid scan mode. Raw MS data were analysed using Progenesis QI for Proteomics software v3.0 (Nonlinear Dynamics). MS/MS spectra were searched against the Swiss-Prot *Homo Sapiens* Reference proteome (retrieved 15/11/2016) using Mascot v2.5.1 (Matrix Science) allowing for a precursor mass tolerance of 10 ppm and a fragment ion tolerance of 0.5 Da. Carbamidomethylation on Cysteines was defined as fixed modification and variable modifications included Deamidation on Asparagine and Glutamine, and Oxidation on Methionine. The peptide FDR was set at 1% and all peptides with an ion score higher than 20 were imported into Progenesis QIP.

### Normalisation and statistical analysis

Proteins that were defined with at least one unique peptide were included in the protein data set for further analysis. Protein abundance values were normalised by centring on the median abundance of the 90% of proteins with the lowest variance across all runs and scaled by median absolute deviation [16]. Missing values were imputed using *k*-nearest neighbours with *k* = 3. Since the normalised log abundance values followed a normal distribution, comparisons of abundance were performed using a Welch’s *t* test with FDR correction using the Benjamini–Hochberg step-up procedure. Longitudinal analysis was performed using linear mixed-effects models with a fixed slope, random intercept model, unstructured covariance structure and degrees of freedom as described by Pinheiro and Bates, for both total EV number and individual proteins identified in proteomic analysis, anchored to the first visit [17]. Variables with a slope estimation that differed from zero *p* < 0.05 (total EV number) or FDR-adjusted-*p* < 0.1 (proteomic data) were considered significant. Principal components analysis (PCA) was performed using normalised, imputed abundance and hierarchical clustering using Euclidian distance. Comparisons of modal EV size and total number were performed using a Mann–Whitney *U* test or Kruskal–Wallis *H* test, since the distribution of values was non-normal.

Gene Ontology (GO) analysis was performed using WebGestalt in R [18]. Overrepresentation analysis was performed using with a foreground–background approach to identify enriched component terms in the proteomic dataset when compared with a comprehensive dataset of the CSF proteome [19]. GO analysis comparing healthy control and ALS samples, and comparing *C9orf72*-associated ALS and non *C9orf72* ALS, was performed in two ways: overrepresentation analysis comparing proteins with unadjusted-*p*-value < 0.05 and log_2_ fold change either > 0.5 or < − 0.5 as the foreground and a background of all proteins identified in this dataset combined with CSF EV proteins identified in two large proteomic datasets [5, 6]; and Gene Set Enrichment Analysis using all proteins identified, ranked by the product of fold change and − log_10_ *p*-value with 1000 permutations. All enrichment analyses underwent Benjamini–Hochberg FDR correction.

## Results

### EV isolation

**Fig. 2:**
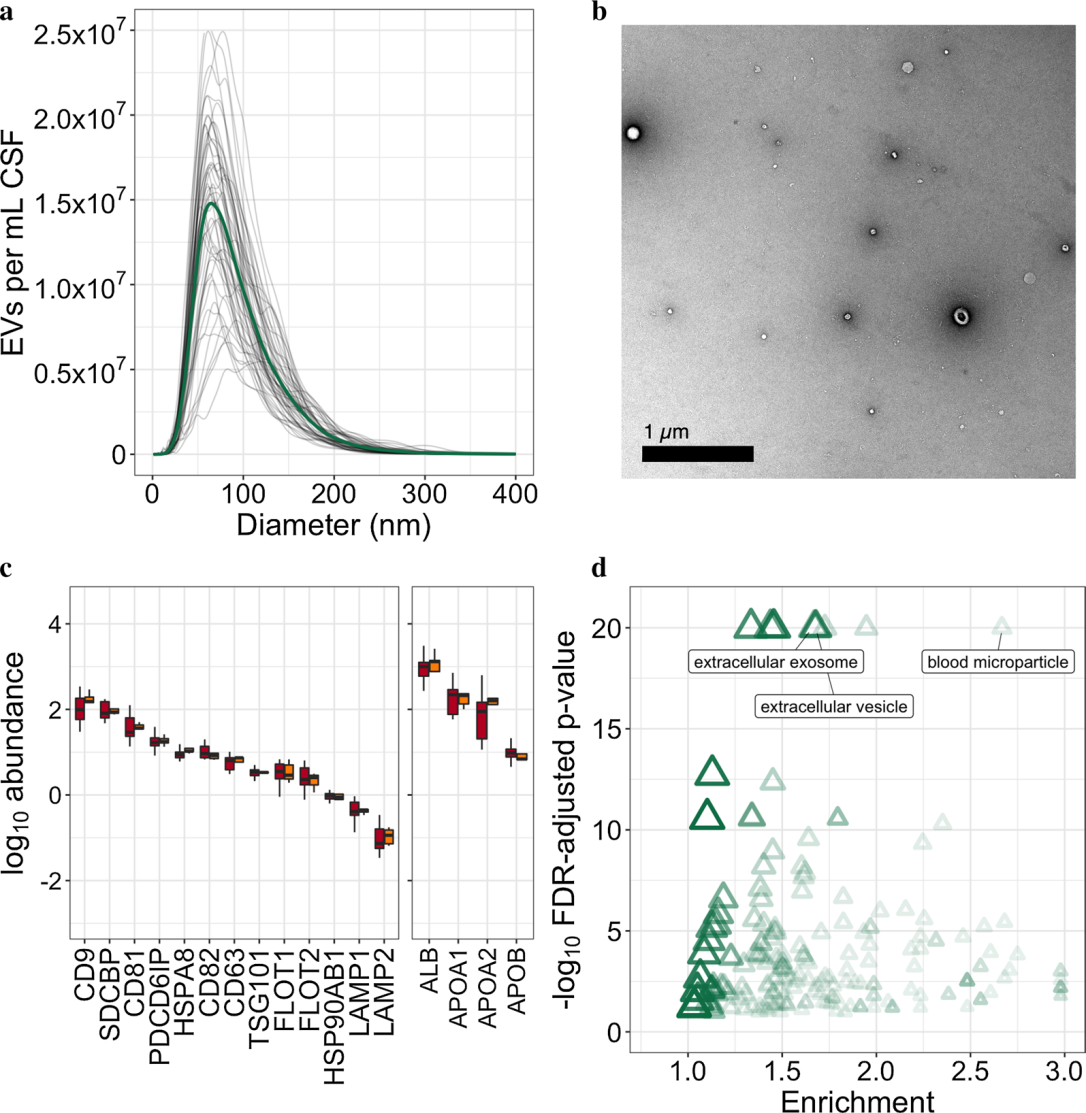
EV characterisation. **a** nanoparticle tracking analysis of extracted CSF EVs (grey lines indicate individual sample particle counts, solid green line indicates mean count for each particle size). **b** Transmission electron micrograph of extracted EVs (obtained from a pooled control sample). **c** EV marker proteins and contaminants (denoted by gene symbol) identified within the proteomic dataset in healthy control (yellow) and ALS first-visit (red) samples. **d** Gene ontology cellular component overrepresentation analysis, using identifications from the CSF proteome resource as the background list, indicates significant enrichment of exosome, microvesicle and extracellular vesicle terms. ALS: amyotrophic lateral sclerosis; EV: extracellular vesicle; FDR: false discovery rate; CSF: cerebrospinal fluid; CD9—UniProt P21926; SDCBP—O00560; CD81—P60033; PDCD6IP—Q8WUM4; HSPA8—P11142; CD82—P27701; CD63—P08962; TSG101—Q99816; FLOT1—O75955; FLOT2—Q14254; HSP90AB1—P08238; LAMP1—P11279; LAMP2—P13473; ALB—P02768; APOA1—P02647; APOA2—P02652; APOB—P04114

UFLC extracted particles with a size distribution consistent with a mixed population of EVs (Fig. 2a). Transmission electron microscopy demonstrated particles with morphology consistent with EVs (Fig. 2b). Proteomic analysis identified numerous surface and luminal EV markers including CD9, Programmed cell death 6-interacting protein (ALIX), Tumour suppressor gene 101, Flotilins 1 and 2 (Fig. 2c). GO overrepresentation analysis, comparing proteins identified in proteomic analysis with a large dataset of the whole CSF proteome [19] indicated significant enrichment of component terms “extracellular exosome” (foreground 547/background 975, OR 1.63, FDR-adjusted *p* < 0.001), “extracellular vesicle” (557/988, OR 1.64, *p* < 0.001) and “blood microparticle” (73/85, OR 2.49, *p* < 0.001; Fig. 2d; top 200 GO component GO terms (by *p*-value) can be found in Additional file 1: Table S1).

### CSF EV size distribution and number are similar in ALS and healthy controls and in ALS patients with and without a *C9orf72* hexanucleotide repeat expansion

**Fig. 3:**
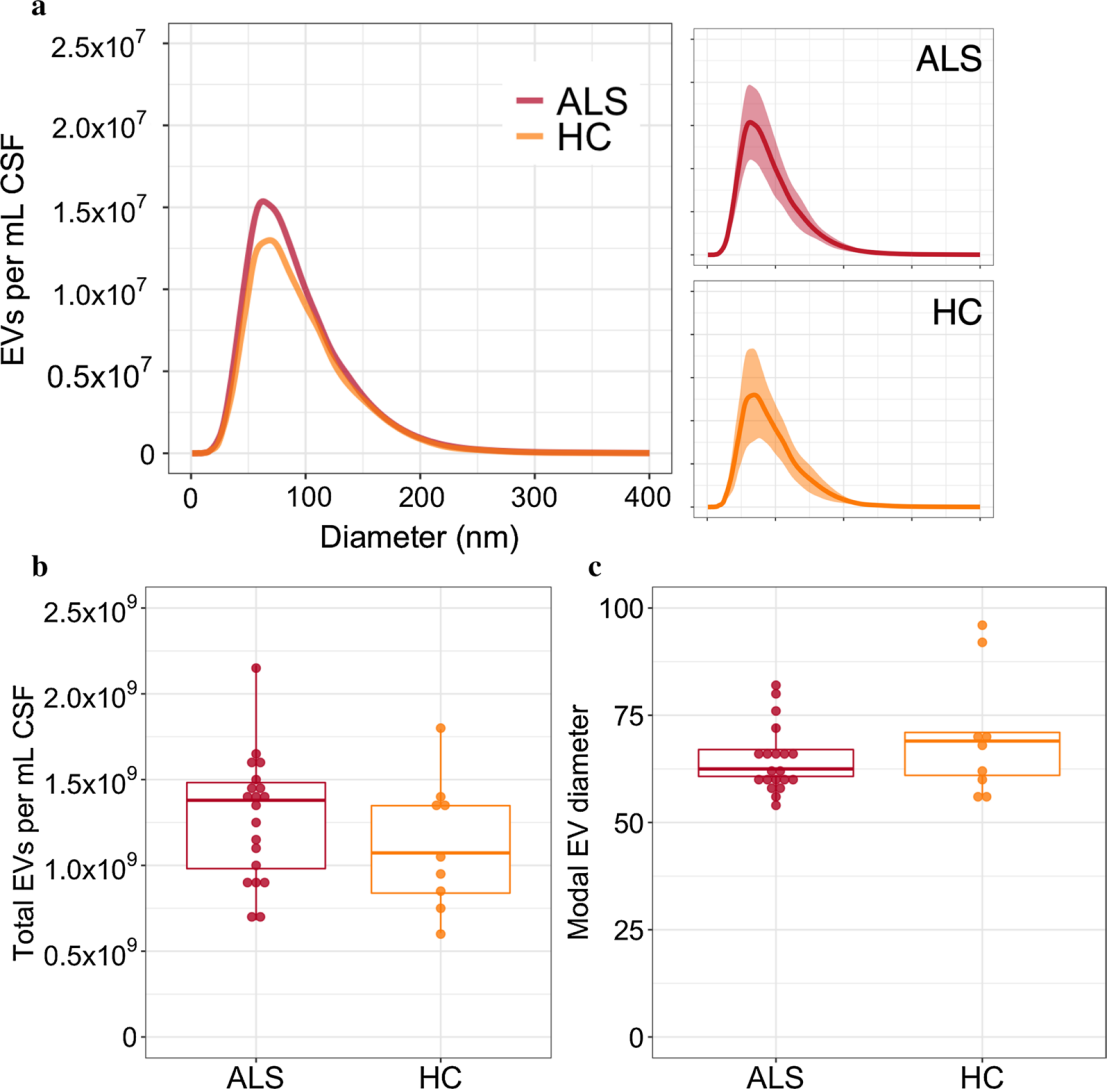
**a** Mean size distribution of CSF EVs in patients with ALS (*n* = 20) and healthy controls (*n* = 9) overlaid. Mean ± SD for groups shown in neighbouring plots. **b** Total number of EVs per mL CSF and **C** modal size of extracted EVs. ALS: amyotrophic lateral sclerosis; EV: extracellular vesicle; CSF: cerebrospinal fluid; HC: healthy control

**Fig. 4:**
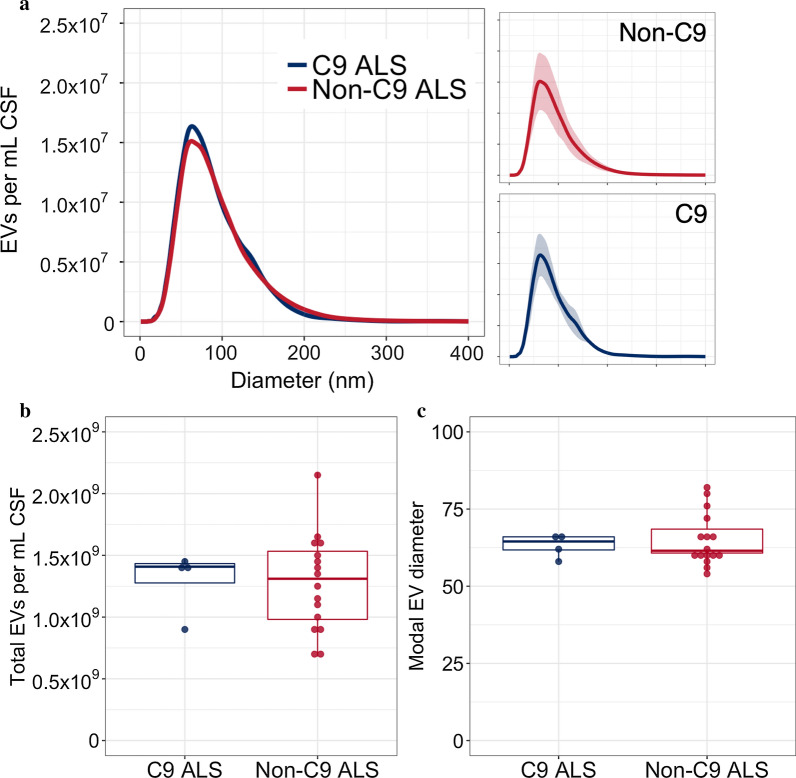
**a** Mean size distribution of CSF EVs in patients with *C9orf72*-associated (*n* = 4) and non-*C9orf72*-associated ALS (*n* = 16) overlaid. Mean ± SD for groups shown in neighbouring plots **b** total number of EVs per mL CSF and **c** modal size of extracted EVs. ALS: amyotrophic lateral sclerosis; EV: extracellular vesicle; CSF: cerebrospinal fluid; C9: *C9orf72* hexanucleotide repeat expansion

Due to reduced EV secretion demonstrated in *C9orf72* iPSCs [11], EV size distribution and number were compared between ALS patients and healthy controls, and ALS patients with and without a *C9orf72* hexanucleotide repeat expansion. No difference in the modal size or total number of EVs isolated per mL starting CSF were found between ALS patients and controls (total number *p* = 0.187, modal size *p* = 0.395; Fig. 3). There was no significant difference comparing the sub-groups of ALS patients with and without a hexanucleotide repeat expansion in *C9orf72* (total number *p* = 0.284, modal size *p* = 0.619; Fig. 4).

The overall variation in EV number per mL CSF was high (CV 30%). There was no correlation of EV number with the cross-sectional disease progression rate at first visit (Pearson’s *r* 0.03, *p* = 0.913) or age at sampling (Pearson’s *r* = 0.18, *p* = 0.437). In addition, there was no longitudinal change in total EV number in the ALS samples for which longitudinal data was available (18 samples from 7 participants; slope = − 0.08 × 10^8^ EV per month, *p* = 0.643).

### The CSF EV proteome in ALS

Proteomic data from 8 ALS patients and 4 healthy controls had to be excluded from subsequent analysis due to a chromatographic failure that developed part way through the experiment, leading to an excessive quantity of missing data (> 50%). The demographic information of the included samples (CSF EVs from 12 ALS patients, including 3 carrying a *C9orf72* hexanucleotide repeat, and 5 healthy controls) is given in Table 2. A total of 1020 proteins were identified and quantified, with only 1.9% missing values (non-normalised intensity data output from Progenesis in Additional file 1: Table S2). The median combined CV across all subject groups for all proteins was 26.7% (IQR 20.3–36.2%). The median CV for pooled samples was 10% (IQR 6–17%; difference between pooled sample and individual sample CVs *p* < 0.001); pairwise correlation between pooled samples median 0.96 (Pearson’s *r*; all *p* < 0.001; IQR 0.95–0.97), non-pooled samples pairwise correlation median 0.86 (IQR 0.78–0.89; difference between pooled sample and individual sample pairwise correlation coefficients *p* < 0.001).

**Table 2:** Demographic data for CSF EV proteomic analysis

	ALS			Healthy control	*p*	
	All	*C9orf72*+	*C9orf72*−		HC vs ALS	*C9orf72 *+ vs non-*C9orf72* −
N	12	3	9	5	–	–
%male	66.7	33.3	77.8	20	0.131^a^	0.236^a^
Age at sampling, mean ± SD (years)	68.1 ± 12.0	51.9 ± 15.6	65 ± 10.0	52.0 ± 14.8	0.113^b^	0.166^b^
Age at onset, mean ± SD (years)	59.7 ± 12.4	51.0 ± 15.6	62.6 ± 10.7	–	–	0.229^b^
% bulbar onset	50.0	66.7	44.4	–	–	0.545^a^
Disease progression rate, median [IQR]	0.53 [0.21–0.63]	0.62 [0.37–0.83]	0.51 [0.22–0.60]	–	–	0.782^b^

*ALS* amyotrophic lateral sclerosis, *HC* healthy control, *SD* standard deviation, *IQR* interquartile range

^a^Fisher exact test. ^b^Kruskal–Wallis *H* test

### Cross sectional analysis demonstrates downregulation of Bleomycin hydrolase in CSF EVs from ALS patients

**Fig. 5:**
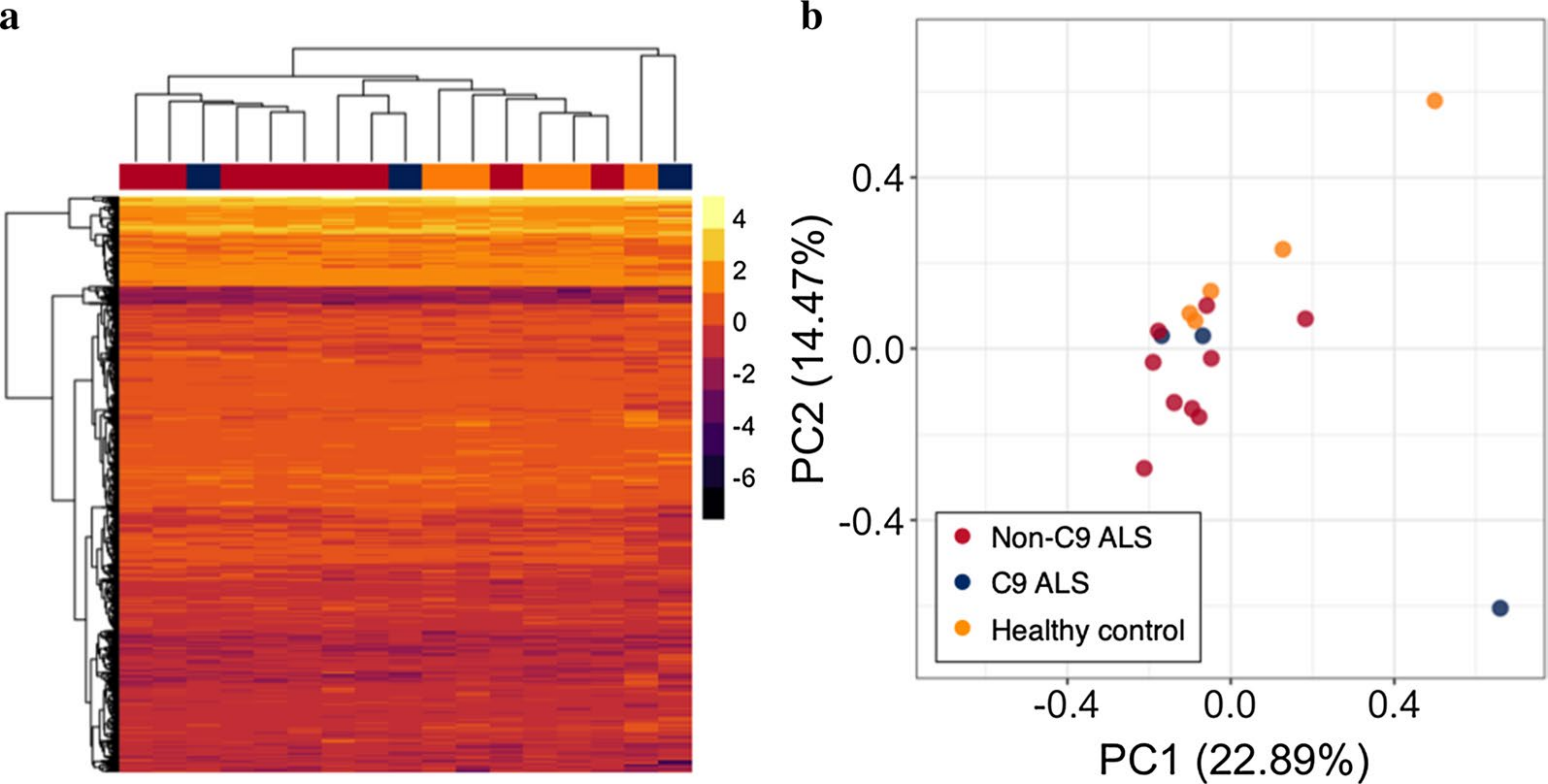
**a** Heatmap and hierarchical clustering of protein abundance in the CSF EV proteome. Column colour bar indicates sample group. **b** PCA biplot of CSF EV samples. ALS: amyotrophic lateral sclerosis; EV: extracellular vesicle; CSF: cerebrospinal fluid; HC: healthy control; PC: principal component; C9: *C9orf72* hexanucleotide repeat expansion

**Fig. 6:**
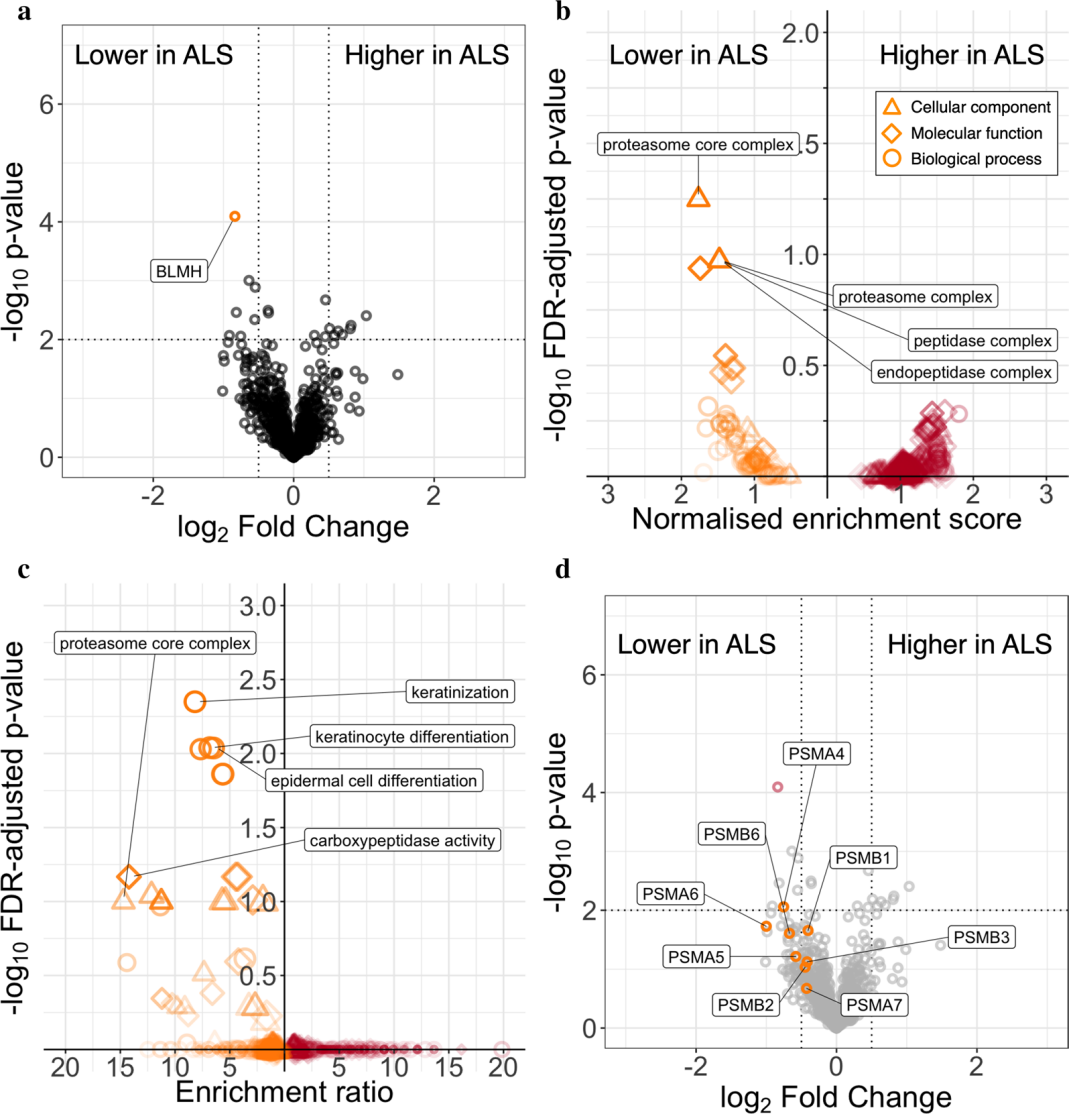
The CSF EV proteome in ALS. **a** Volcano plot of the CSF EV proteome in ALS (*n* = 12, first visit samples only) compared with healthy control subjects (*n* = 5). Highlighted points indicate FDR-adjusted *p* < 0.1. Negative log fold change indicates proteins downregulated in ALS compared with controls. Dotted lines indicate *p* < 0.01 and log_2_ fold change ± 0.5. **b** Volcano plot of Gene Ontology (GO) terms by gene set enrichment analysis, ordered by the product of -log_10_ *p*-value and fold change. GO terms with FDR-adjusted *p* < 0.1 labelled. **c** Volcano plot of GO overrepresentation analysis of proteins upregulated or downregulated in ALS compared with healthy controls, *p* < 0.05 and log fold change > 0.5 or < − 0.5 compared with healthy controls. Selected GO terms labelled. **d** Volcano plot of proteomic analysis data, proteins annotated to “proteasome core complex” labelled and highlighted. ALS: amyotrophic lateral sclerosis; EV: extracellular vesicle; CSF: cerebrospinal fluid; HC: healthy control; BLMH: Bleomycin hydrolase (UniProt Q13867); PSMA: Proteasome alpha subunit (PSMA4—P25789; PSMA5—P28066; PSMA6—P60900; PSMA7—O14818); PSMB: Proteasome beta subunit (PSMB1—P20618; PSMB2—P49721; PSMB3—P49720; PSMB6—P28072); GO: Gene ontology

Hierarchical clustering and PCA of first-visit samples did not lead to separation into subject groups (Fig. 5), suggesting that most variance in the data is not attributable to subject group. Comparison of CSF EV protein abundance between ALS patients and healthy controls identified downregulation of one protein, Bleomycin hydrolase (BLMH, Q13867), in ALS patients (FDR-adjusted *p* < 0.1; Fig. 6a).

### GO enrichment analysis indicates downregulation of proteasomal terms in ALS patient CSF EVs

GSEA identified significant diminution (FDR-adjusted *p* < 0.1) of “proteasome core complex” (8/8 genes normalized enrichment ratio − 1.77, FDR-adjusted *p* = 0.057). Other proteasomal terms were also similarly decreased, though did not meet the < 0.1 FDR threshold (e.g. “endopeptidase complex”, ratio − 1.48, FDR-adjusted *p* = 0.107; Fig. 6b and Additional file 1: Table S3). Similarly, overrepresentation analysis (Fig. 6c and Additional file 1: Table S4) identified GO terms related to the proteasome (e.g. “peptidase activity, acting on L-amino acid peptides”, 7/97 proteins, odds ration − 4.44, FDR-adjusted *p* = 0.068; “carboxypeptidase activity” 3/13 proteins, OR − 14.21, FDR-adjusted *p* = 0.068; “proteasome core complex” 3/13 proteins, OR − 14.21, FDR-adjusted *p* = 0.100) as well as terms related to keratinisation (e.g. “keratinization”, 8/61 proteins, OR − 8.18, FDR-adjusted *p *= 0.004) in proteins downregulated in ALS. There was no GO term enrichment in proteins upregulated in ALS CSF EVs.

All proteins in the proteomic dataset encoded by genes annotated to the cellular component term “proteasome core complex” i.e. proteasomal alpha (PSMA4 UniProt P25789; PSMA5, P28066; PSMA6, P60900; PSMA7, O14818) and beta subunits (PSMB1, P20618; PSMB2, P49721; PSMB3, P49720; PSMB6, P28072), had negative log fold change in ALS compared with healthy controls (Fig. 6d).

### No temporal changes are identified in longitudinal analysis of ALS samples

Longitudinal analysis of EV proteins was performed to determine whether there were detectable temporal changes in the CSF proteome during ALS in a subset of ALS patients (n = 5 participants, 2–3 samples per participant). This did not identify any proteins with significant increase or decrease over time (FDR-adjusted *p* < 0.1).

### Univariate analysis of C9orf72 ALS vs non-C9orf72 ALS

**Fig. 7:**
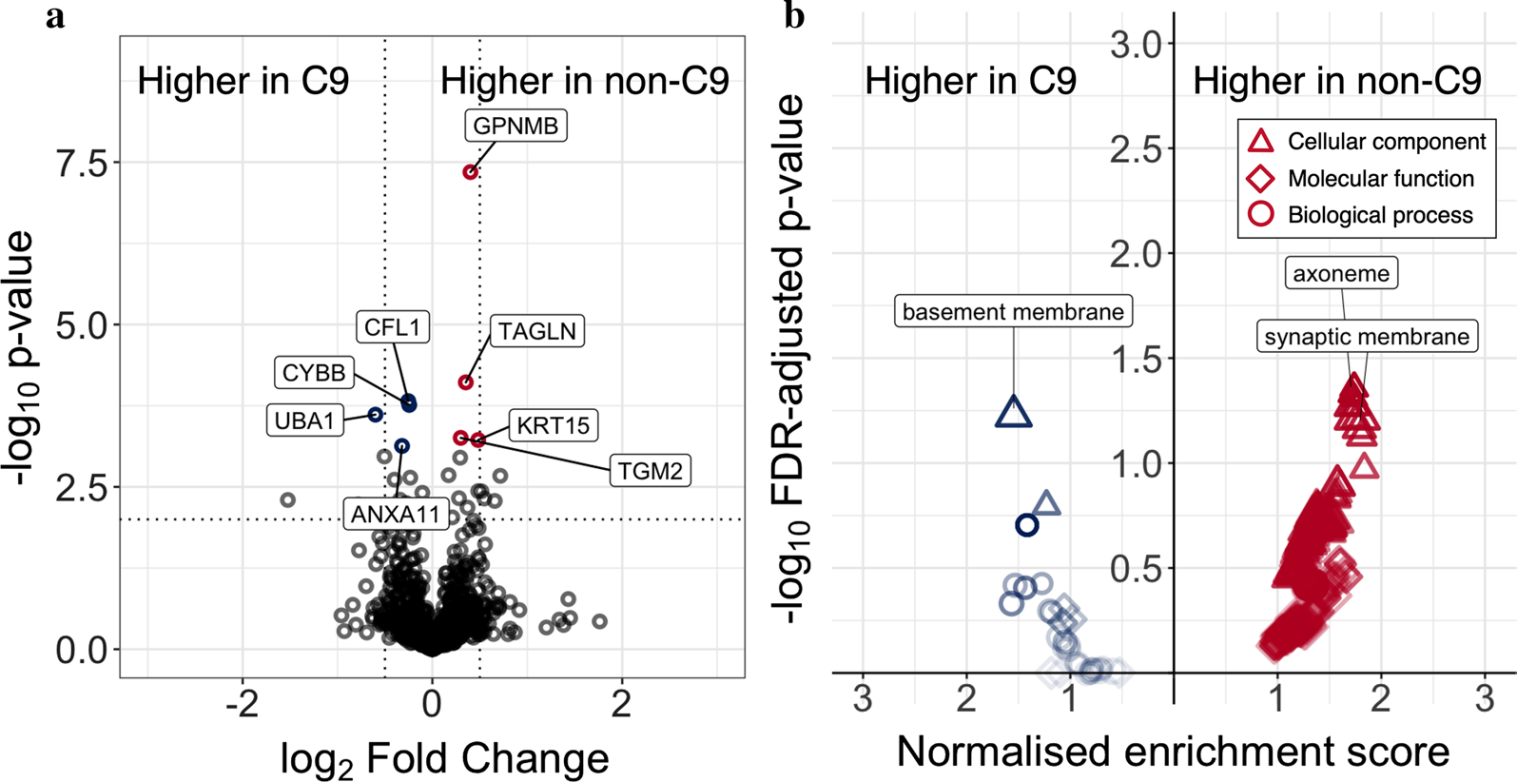
**a** Volcano plot of the CSF EV proteome in *C9orf72* ALS (n = 3) compared with non-*C9orf72* ALS subjects (n = 9, first-visit samples only). Red points indicate FDR-adjusted *p* < 0.1, higher in non-*C9orf72* ALS, blue points FDR-adjusted *p* < 0.1 higher in *C9orf72* ALS. Negative log fold change indicates proteins downregulated in non-*C9orf72* ALS compared with *C9orf72* ALS. Dotted lines indicate *p* < 0.01 and log_2_ fold change ± 0.5. **b** Gene ontology (GO) gene set enrichment analysis of the CSF EV proteome in *C9orf72* ALS compared with non-*C9orf72* ALS. Representative up- and down-regulated terms labelled. ALS: amyotrophic lateral sclerosis; EV: extracellular vesicle; CSF: cerebrospinal fluid; GO: gene ontology; C9: *C9orf72* hexanucleotide repeat expansion-associated ALS; CFL1—UniProt P23528; CYBB—P04839; UBA1—P22314; ANXA11—P50955; GPNMB—Q14956; TAGLN—Q01995; TGM2—P21980; KRT13—P13646

Protein abundance was compared between ALS patients carrying a *C9orf72* hexanucleotide repeat expansion (n = 3) and ALS patients without an expansion (n = 9). Six proteins were differentially abundant in the CSF EV proteome in the sub-group of ALS associated with the *C9orf72* hexanucleotide repeat (FDR-adjusted *p* < 0.1), including upregulation of Transmembrane glycoprotein NMB (GPNMB, Q14956) and Protein-glutamine gamma-glutamyltransferase 2 (TGM2, P21980), and downregulation of Annexin 11 (ANXA11, P50995), Ubiquitin-like modifier-activating enzyme 1 (UBA1, P22314), Cytochrome b-245 heavy chain (CYBB P04839), Cofilin-1 (CFL1; Fig. 7a). Only UBA1, a protein involved in ubiquitination of proteins for degradation by the UPS, had a log_2_ fold change of > 0.5.

### GO enrichment analysis indicates limited differences between the C9orf72 ALS and non-C9orf72 ALS CSF EV proteomes

Seeking systematic alterations in the CSF EV proteome between *C9orf72*-associated and non-*C9orf72*-associated ALS, GO enrichment analysis was performed by way of overrepresentation analysis and gene set enrichment analysis. There was upregulation of terms related to the synaptic membrane and axoneme in non-*C9orf72*-associated ALS compared with *C9orf72*-associated ALS (e.g. “integral component of postsynaptic membrane”, 8/10 genes, normalized enrichment ratio 1.74, FDR-adjusted *p* = 0.044; “axoneme”, 6/10 proteins, ratio 1.71 FDR-adjusted *p* = 0.047; Fig. 7B and Additional file 1: Table S5). The only term with significant enrichment in *C9orf72*-associated ALS was “basement membrane” (41/41 genes, ratio -1.55, FDR-adjuste *p* = 0.059). This was not recapitulated in GO overrepresentation analysis, which identified no significant overrepresentation of GO terms in favouring *C9orf72*-associated or non-*C9orf72*-associated ALS CSF EVs (Additional file 1: Table S6).

## Discussion

We have shown that it is possible to detect disease-relevant changes in the proteome of EVs in ALS, despite the considerable technical challenges caused by their low abundance in CSF, thus laying the foundation for further exploration of EVs as a source of biomarkers. The study of size distribution using NTA did not demonstrate differences in the total number or modal size of EVs purified from the CSF of patients with ALS (including when subdivided by *C9orf72* status). We have previously reported a significant deficit in EV production from fibroblasts and iPSC motor neurons derived from ALS patients with a *C9orf72* hexanucleotide repeat expansion [11]. The current finding may be a reflection of the multiple cell types that contribute to the CSF EV population (such as oligodendrocytes and choroid plexus [5]), or might suggest that the mechanisms leading to altered EV secretion in *C9orf72* expansion-carrying cells in vitro do not accurately recapitulate events in vivo. The high variability of the CSF EV concentration was also notable, varying from 0.5 to 2.2 × 10^9^ per mL CSF. Although this could lead to reduced power to detect a significant difference, the median total EV number per mL CSF was extremely similar between ALS and control groups. There was also no correlation with the rate of disease progression or with age (a decline in CSF EV concentration with increasing age was noted in a previous study [20]).

Analysis of the EV proteome yielded good proteomic depth, with over 1200 protein groups identified, though the list of candidate markers identified that were differentially abundant between ALS and healthy controls, using a FDR threshold of 10% and log_2_ fold change of 0.5 was small: just one protein, BLMH, when considering only first-visit ALS samples. BLMH forms a hexameric barrel-shaped protease somewhat homologous to the proteasome [21], with diverse proteolytic activity [22]. BLMH has been implicated in Huntington’s disease, through cleavage of huntingtin [23], and in Alzheimer’s disease through the processing of amyloid precursor protein [24]. Although clearly requiring validation, BLMH has biological plausibility in ALS pathogenesis.

There are several potential reasons for this low number of identified candidates. The relatively high variability observed (median combined CV across the cohort of > 29% and 50% in the lowest intensity proteins) will have contributed, along with the small sample size due to exclusion of samples with large quantities of missing data.

The main signal emerging from GO enrichment analysis suggests involvement of proteostatic mechanisms was detectable in CSF EVs in ALS, primarily through downregulation of proteasomal proteins. Corroborating this finding, a recent study of CSF exosomal mRNA patients also found downregulation of transcripts associated with the ubiquitin proteasome system in ALS [25]. Protein homeostasis is implicated in the pathogenesis of ALS and other neurodegenerative diseases, with mutations in genes encoding both UPS and autophagy proteins associated with the development of ALS [26]. There is also pathological evidence to support reduced proteasomal activity and decreases in 20S proteasomal subunit levels in spinal cord, along with reduced staining for 20S subunits in ventral horn motor neurons of patients with ALS [27]; this is somewhat controversial, as other work has demonstrated increased proteasomal staining in ALS [28]. Upregulation of autophagy is implied by p62 immunopositivity of neuronal and glial inclusions [29, 30]. Autophagosomes, though absent from spinal motor neurons in non-ALS pathological specimens, have been observed in both normal-appearing and degenerating spinal motor neurons in ALS, implying upregulation of autophagy [31].

Although analysis in *C9orf72*-expansion associated ALS yielded more significant proteins at the 10% FDR level, these were of low fold change and GO enrichment analysis demonstrated limited systematic changes in the proteome, predominantly hinting at differences in synaptic membrane proteins between *C9orf72* and non-*C9orf72* ALS. The small number of *C9orf72* positive samples will have impacted power in this analysis and highlights the need for further study in this group.

Though the findings of this study are corroborated by previously published data in in CSF exosomal mRNA and pathological data from *post mortem* tissue [25, 27], further validation is required in larger cohorts to understand the clinical associations and robustness of these specific findings. One previous study of the CSF EV proteome in ALS did not detect these changes, though only 334 proteins were identified in that analysis and only univariate comparison of ALS and control proteomes was performed without exploring systematic differences within the entire proteome [14].

## Conclusion

Detectable alteration in protein homeostatic pathways in CSF EVs offer significant potential in understanding key pathways in ALS pathogenesis, which might form the basis for biomarker development and therapeutic targeting.

## Supplementary information

**Additional file 1: Table S1.** Top 200 GO terms overrepresented in extracted EV proteome compared with the whole CSF proteome as identified in the CSF proteome resource. **Table S2.** Progenesis quantificaion and identifications data (non-normalised, non-transformed). **Table S3.** GO term enrichment using gene set enrichment analysis comparing ALS and healthy control (positive score indicates enrichment in upregulated proteins in ALS). **Table S4.** GO term overrepresentation analysis comparing ALS and healthy control (positive score indicates enrichment in upregulated proteins in ALS). **Table S5.** GO term enrichment using gene set enrichment analysis comparing ALS and *C9orf72* positive patients (positive score indicates enrichment in upregulated in non *C9orf72*-associated ALS). **Table S6.** GO term overrepresentation analysis comparing ALS and C9orf72 positive patients (positive score indicates enrichment in upregulated proteins in non *C9orf72*-associated ALS).

## Data Availability

The dataset supporting the conclusions of this article is included within the article and its additional file. The mass spectrometry proteomics data have been deposited to the ProteomeXchange Consortium via the PRIDE partner repository with the dataset identifier PXD020499 and 10.6019/pxd020499.

## References

[1] Talbot K, Feneberg E, Scaber J, Thompson AG, Turner MR (2018). Amyotrophic lateral sclerosis: the complex path to precision medicine. J Neurol.

[2] Verber NS, Shepheard SR, Sassani M, McDonough HE, Moore SA, Alix JJP (2019). Biomarkers in motor neuron disease: a state of the art review. Front Neurol.

[3] Zou ZY, Zhou ZR, Che CH, Liu CY, He RL, Huang HP (2017). Genetic epidemiology of amyotrophic lateral sclerosis: a systematic review and meta-analysis. J Neurol Neurosurg Psychiatry.

[4] EL Andaloussi S, Mäger I, Breakefield XO, Wood MJA (2013). Extracellular vesicles: biology and emerging therapeutic opportunities. Nat Rev Drug Discov..

[5] Thompson AG, Gray E, Mager I, Fischer R, Thézénas ML, Charles PD (2018). UFLC-derived CSF extracellular vesicle origin and proteome. Proteomics.

[6] Chiasserini D, Van Weering JRT, Piersma SR, Pham TV, Malekzadeh A, Teunissen CE (2014). Proteomic analysis of cerebrospinal fluid extracellular vesicles: a comprehensive dataset. J Proteomics..

[7] Feiler MS, Strobel B, Freischmidt A, Helferich AM, Kappel J, Brewer BM (2015). TDP-43 is intercellularly transmitted across axon terminals. J Cell Biol.

[8] Murray ME, DeJesus-Hernandez M, Rutherford NJ, Baker M, Duara R, Graff-Radford NR (2011). Clinical and neuropathologic heterogeneity of c9FTD/ALS associated with hexanucleotide repeat expansion in C9ORF72. Acta Neuropathol.

[9] Thompson AG, Gray E, Heman-Ackah SM, Mager I, Talbot K, Andaloussi SE (2016). Extracellular vesicles in neurodegenerative disease—pathogenesis to biomarkers. Nat Rev Neurol.

[10] Iguchi Y, Eid L, Parent M, Soucy G, Bareil C, Riku Y (2016). Exosome secretion is a key pathway for clearance of pathological TDP-43. Brain.

[11] Aoki Y, Manzano R, Lee Y, Dafinca R, Aoki M, Douglas AGL (2017). C9orf72 and RAB7L1 regulate vesicle trafficking in amyotrophic lateral sclerosis and frontotemporal dementia. Brain.

[12] Feneberg E, Steinacker P, Lehnert S, Schneider A, Walther P, Thal DR (2014). Limited role of free TDP-43 as a diagnostic tool in neurodegenerative diseases. Amyotroph Lateral Scler Frontotemporal Degener.

[13] Ding X, Ma M, Teng J, Teng RKF, Zhou S, Yin J (2015). Exposure to ALS-FTD-CSF generates TDP-43 aggregates in glioblastoma cells through exosomes and TNTs-like structure. Oncotarget..

[14] Hayashi N, Doi H, Kurata Y, Kagawa H, Atobe Y, Funakoshi K (2019). Proteomic analysis of exosome-enriched fractions derived from cerebrospinal fluid of amyotrophic lateral sclerosis patients. Neurosci Res.

[15] Théry C, Witwer KW, Aikawa E, Alcaraz MJ, Anderson JD, Andriantsitohaina R (2018). Minimal information for studies of extracellular vesicles 2018 (MISEV2018): a position statement of the International Society for Extracellular Vesicles and update of the MISEV2014 guidelines. J Extracell Vesicles..

[16] Keilhauer EC, Hein MY, Mann M (2015). Accurate protein complex retrieval by affinity enrichment mass spectrometry (AE-MS) rather than affinity purification mass spectrometry (AP-MS). Mol Cell Proteomics.

[17] Pinheiro JC, Bates DM (2000). Mixed-effects models in S and S-PLUS.

[18] Liao Y, Wang J, Jaehnig EJ, Shi Z, Zhang B (2019). WebGestalt 2019: gene set analysis toolkit with revamped UIs and APIs. Nucleic Acids Res.

[19] Guldbrandsen A, Vethe H, Farag Y, Oveland E, Garberg H, Berle M (2014). In-depth characterization of the cerebrospinal fluid (CSF) proteome displayed through the CSF proteome resource (CSF-PR). Mol Cell Proteomics.

[20] Tietje A, Maron KN, Wei Y, Feliciano DM (2014). Cerebrospinal fluid extracellular vesicles undergo age dependent declines and contain known and novel non-coding RNAs. PLoS ONE..

[21] O’Farrell PA, Gonzalez F, Zheng W, Johnston SA, Joshua-Tor L (1999). Crystal structure of human bleomycin hydrolase, a self- compartmentalizing cysteine protease. Structure..

[22] Zheng W, Johnston SA, Joshua-Tor L (1998). The unusual active site of Gal6/bleomycin hydrolase can act as a carboxypeptidase, aminopeptidase, and peptide ligase. Cell.

[23] Ratovitski T, Chighladze E, Waldron E, Hirschhorn RR, Ross CA (2011). Cysteine proteases bleomycin hydrolase and cathepsin Z mediate N-terminal proteolysis and toxicity of mutant huntingtin. J Biol Chem.

[24] Lefterov IM, Koldamova RP, Lazo JS (2000). Human bleomycin hydrolase regulates the secretion of amyloid precursor protein. FASEB J..

[25] Otake K, Kamiguchi H, Hirozane Y (2019). Identification of biomarkers for amyotrophic lateral sclerosis by comprehensive analysis of exosomal mRNAs in human cerebrospinal fluid. BMC Med Genomics.

[26] Taylor JP, Brown RHJ, Cleveland DW (2016). Decoding ALS: from genes to mechanism. Nature.

[27] Kabashi E, Agar JN, Strong MJ, Durham HD (2012). Impaired proteasome function in sporadic amyotrophic lateral sclerosis. Amyotroph Lateral Scler.

[28] Mendonça DMF, Chimelli L, Martinez AMB (2006). Expression of ubiquitin and proteasome in motorneurons and astrocytes of spinal cords from patients with amyotrophic lateral sclerosis. Neurosci Lett.

[29] Arai T, Nonaka T, Hasegawa M, Akiyama H, Yoshida M, Hashizume Y (2003). Neuronal and glial inclusions in frontotemporal dementia with or without motor neuron disease are immunopositive for p62. Neurosci Lett.

[30] Mizuno Y, Amari M, Takatama M, Aizawa H, Mihara B, Okamoto K (2006). Immunoreactivities of p62, an ubiqutin-binding protein, in the spinal anterior horn cells of patients with amyotrophic lateral sclerosis. J Neurol Sci.

[31] Sasaki S (2011). Autophagy in spinal cord motor neurons in sporadic amyotrophic lateral sclerosis. J Neuropathol Exp Neurol.

